# Aerobic training as a means to enhance inhibition: what’s yet to be studied?

**DOI:** 10.1186/s11556-015-0160-9

**Published:** 2015-12-24

**Authors:** Oron Levin, Yael Netz

**Affiliations:** Movement Control and Neuroplasticity Research Group, Department of Kinesiology, Group Biomedical Sciences, KU Leuven Tervuursevest 101, bus 1501, B-3001 Leuven, Belgium; Zinman College for Physical Education and Sport Sciences, Wingate Institute, Netanya, Israel

**Keywords:** Aging, Inhibition, Aerobic exercise training, Brain neurotransmitters, Brain networks, Motor control

## Abstract

Some of the neurodegenerative processes in healthy aging, including changes in structural and biochemical properties of the brain, are argued to affect cortical inhibitory functions. Age-related deficits in the ability to control cerebral inhibition may explain wide range of motor and cognitive deficits that healthy older adults experience in daily life such as impaired coordination skills and declines in attention, concentration, and learning abilities. Importantly, evidence from many studies suggests that impaired inhibitory control in advancing age can be delayed or even alleviated by aerobic exercise training. Findings from a recent study by Duchesne and colleagues (2015) may provide insights into this process. First, observations from Duchesne et al. indicated that aerobic exercise training program improved cognitive inhibitory functioning in both patients with Parkinson’s disease (PD) and matched older controls. Second, Duchesne et al. showed that cognitive inhibition and motor skills were highly correlated both pre- and post-exercise in PD but not in controls. Based on the aforementioned findings we highlight possible mechanisms that may play a role in the interactions between cognitive and motor inhibitory functions in healthy elderly that could benefit from aerobic exercise training: specifically, the brain neurotransmission systems and the frontal-basal ganglia network. In conclusion, we raise two fundamental questions which are yet to be addressed: (1) the extent to which different brain neurotransmitter systems are affected by aerobic exercise training; (2) the extent to which neurotransmitter levels prior to the onset of intervention may facilitate (or impede) training-induced neuroplasticity in the aging brain.

## Background

Inhibition plays a critical role in control of many cognitive and motor functions [1–3]. Cognitive inhibition can be conceptualized as a process that blocks the spread of activation, keeping attention focused sharply on the task at hand [2]. Motor inhibition is required during withdrawing, cancelation, or selection of voluntary movements [1, 3]. Although distinct, cognitive and motor inhibitory functions are mediated by overlapping prefrontal brain networks [4, 5] which are compromised by aging processes to a greater extent than other regions of the brain [6]. Over the last two decades evidence has accumulated regarding the beneficial effect of aerobic exercise training on brain functions and inhibitory control in elderly [7–12]; see reviews [13–15]. The possibility that aerobic exercise training may lead to more efficient inhibitory processes is of particular interest, given that degraded ability for inhibitory control is a primary cause of declined performance in both cognitive and motor tasks in older age [16, 17]; see reviews [18, 19].

The beneficial effect of aerobic exercise training on both cognitive and motor functioning has been recently examined by Duchesne and colleagues [20] on group of patients with Parkinson’s disease (PD) and healthy older adults. Findings from this study indicate that besides improving physical fitness, the 3-months aerobic exercise training improved cognitive functioning and motor learning skills in both groups; particularly, enhancing participant’s inhibition capacity. For PDs, the baseline performance was differentially associated with training-related improvements in both cognitive and motor domains in the post-tests. While the study did not focus specifically on inhibitory functions, its findings offer a platform for understanding the mutual effect of aerobic exercise training on cognitive and motor functioning in elderly as well as their relationships. In the present commentary we focus, specifically, on neurophysiological mechanisms associated with age-related changes in the regulation of inhibition and discuss a multimodal approach to further our knowledge on the influence of aerobic exercise training on (motor) behavior.

### The neurophysiological basis of age-related changes in inhibitory control

Aging gives rise to structural and neurochemical changes in the central nervous system (CNS) that could impair connectivity within and between local and distributed brain networks, leading to cognitive and motor declines [21, 22]. Structural changes are normally characterized by a decrease in white matter microstructural organization [21–25] and gray matter loss [26–28]. Structural declines may occur in parallel with depletion in the concentrations of regional levels of neurotransmitters such as gamma-aminobutyric acid (GABA) [29, 30] and serotonin [31–35]; see review [36]. Deficiencies in GABAergic activity [37–39] and impaired interactions between GABAergic and cholinergic system [40, 41] have been documented, for example, in healthy aging and older adults with mild cognitive impairments who also demonstrated deficient motor inhibition; see review [19].

Movement control requires integration of information from various sensory sources (vision, proprioception, tactile) which is critically dependent on the balance between excitatory and inhibitory processes in the brain. This balance is expected to be perturbed by impaired activation of the above mentioned neurotransmission systems [34, 39] or disruption in connectivity between specific brain substructures [21, 22]. While all healthy older individuals tend to present general declines in both structural and biochemical properties of the brain [21–25, 29, 32, 33, 37–41], performance declines may still not be evident in some individuals due to compensatory recruitment of alternative brain resources [17, 42–44]. The conditions and functions of principal brain neurotransmitter systems and their age- and/or pathology-related alterations can be studied via application of a multimodal approach that combines an array of brain imaging and brain stimulation techniques [15, 19]. For example, assessment of neurochemical properties (i.e., neurotransmitter concentrations and concentration ratios) in specific brain regions can be monitored with magnetic resonance spectroscopy (MRS) [29, 39] or positron emission tomography (PET) [32] whereas the effects of local neurotransmitter levels for cortical inhibitory processes and neurophysiological assessments of brain network activity and connectivity can be studied with functional magnetic resonance imaging (fMRI) and/or transcranial magnetic stimulation [16, 17, 37–44]; see reviews [18, 19, 45].

### The prefrontal-basal ganglia network

Inhibitory functions are believed to be largely subserved by the frontal lobes of the brain. The role of these brain regions in the inhibitory control of action and cognition is well documented [46, 47]; see reviews [1, 48]). Notably, parts of the brain network that regulate inhibition are located in the prefrontal cortex which is more prone to age-related structural changes than posterior areas [23]. Evidence for the involvement of the prefrontal-basal ganglia network in cognitive and motor functioning has been demonstrated by Duchesne et al. [20], showing positive relationship between cognitive and motor abilities in PD individuals. Indeed, recent structural imaging studies clearly show that age-related changes in the microstructural organization of the prefrontal-basal ganglia network are associated with a reduced ability to actively prevent movements when a “stop” is delivered after a “go” [49, 50]. Crucially, age-related changes in brain activity and connectivity are often seen in prefrontal brain regions such as the dorsolateral prefrontal cortex (DLPFC), inferior frontal cortex (IFC), and/or pre-supplementary motor area (pre-SMA) [42, 43] that are classically involved in the suppression of prepotent response tendencies.

Another interesting finding from the study of Duchesne and colleagues [20] was that baseline cognitive performance of PD individuals was positively associated with training-related improvements in both cognitive flexibility and inhibition. This observation suggests that the ability of PDs to improve their motor and/or cognitive skills is determined, largely, by the integrity of the prefrontal network. Taken together, observations from Duchesne et al. [20] call for further investigation of the effect of aerobic exercise training on the functioning of the aging prefrontal-basal ganglia network.

## Conclusions

Duchesne et al. [20] proposed that *“aerobic exercise training can be a valuable non-pharmacological intervention to promote physical fitness in early PD, but also better cognitive and procedural functioning”.* Their findings suggest that aerobic exercise training can at least partly improve and/or restore functionality of dopaminergic system in patients with PD and prompt questions about the effect of aerobic exercise training on the interactions between cognitive and motor functioning. Principal challenges for future research at this juncture are: (1) to examine how other brain neurotransmitter systems are affected by aerobic exercise training; (2) to investigate the extent to which long-term effects of aerobic exercise training on cognitive and motor inhibitory functions (and their interactions) are determine by the integrity of prefrontal neurotransmitter systems; specifically but not exclusively: GABA, serotonin, and dopamine. The addition of a neurochemical perspective to the study of brain function and neuroplasticity is highly innovative and would allow a deeper understanding of mechanisms by which aerobic exercise training acts on cerebral inhibitory processes.

Another principal challenge is to examine whether beneficial effects on cognitive/motor functioning could be further enhanced by combining aerobic exercise training with other types interventions such as pharmacological interventions [30], or non-invasive brain stimulation techniques [51]; the latter may be used to target specific brain structures or neurotransmitter systems. Finally, the fact that older adults can improve inhibitory control through a multiple exercise intervention (i.e., aerobic exercise training combined with the practice of motor/cognitive tasks) even more than through a single exercise intervention (i.e., aerobic exercise training alone) is well documented [52–55]. Future research should work to established to what extent these interventions affect brain regions or networks that regulate inhibition (in association with their effects on motor functioning, cognitive functioning, and health [56]). A critical aim in this respect is to explore the specific effects of each intervention (or combination of interventions) on brain-behavior relationships.
